# Development of a *toxR*-based loop-mediated isothermal amplification assay for detecting *Vibrio parahaemolyticus*

**DOI:** 10.1186/1471-2180-10-41

**Published:** 2010-02-10

**Authors:** Siyi Chen, Beilei Ge

**Affiliations:** 1 Department of Food Science, Louisiana State University Agricultural Center, Baton Rouge, Louisiana 70803, USA

## Abstract

**Background:**

*Vibrio parahaemolyticus *is a leading cause of seafood-related bacterial gastroenteritis and outbreaks worldwide. Sensitive and specific detection methods are needed to better control *V. parahaemolyticus *infections. This study aimed at developing a highly specific and sensitive loop-mediated isothermal amplification (LAMP) assay for detecting *V. parahaemolyticus *in oysters. A set of five LAMP primers, two outer, two inner, and one loop were designed based on the published *V. parahaemolyticus toxR *sequence. Specificity of the assay was evaluated using a panel of 36 *V. parahaemolyticus *and 39 other strains. The assay sensitivity was determined using serial dilutions of *V. parahaemolyticus *ATCC 27969 culture ranging from 10^8 ^CFU/ml to extinction. The assay was also tested in experimentally inoculated oyster samples.

**Results:**

The *toxR*-based LAMP assay was able to specifically detect all of the 36 *V. parahaemolyticus *strains without amplification from 39 other strains. The detection limit was 47-470 cells per reaction in pure culture, up to 100-fold more sensitive than that of *toxR*-PCR. When applied in spiked oysters, the assay was able to detect 1.1 × 10^5 ^*V. parahaemolyticus *cells per gram of oyster without enrichment, up to 100-fold more sensitive than that of *toxR*-PCR. Standard curves generated for detecting *V. parahaemolyticus *in both pure culture and spiked oyster samples showed good linear relationship between cell numbers and the fluorescence or turbidity signals.

**Conclusions:**

The *toxR*-based LAMP assay developed in this study was sensitive, specific, and quantitative, holding great potential for future field detection of *V. parahaemolyticus *in raw oysters.

## Background

The Gram-negative, halophilic marine bacterium *Vibrio parahaemolyticus *has emerged as a major cause of seafood-associated outbreaks throughout the world and become a significant concern of seafood safety [1-3]. Shellfish, particularly oysters, has been frequently implicated in *V. parahaemolyticus *infections [4,5]. Typically within 24 h after eating contaminated seafood, *V. parahaemolyticus *causes acute, self-limiting gastroenteritis characterized by diarrhea, abdominal cramps, nausea, vomiting, fever, and chills, which lasts for 1-3 days [6]. Two hemolysins, the thermostable direct hemolysin (TDH) and the TDH-related hemolysin (TRH) are well-characterized virulence factors for pathogenic *V. parahaemolyticus *strains [7]. However, the majority of *V. parahaemolyticus *strains in the environment and seafood samples lack these two hemolysin genes [8-10], thus the number of total *V. parahaemolyticus *has been used as an indicator for preventing *V. parahaemolyticus *infections from seafood consumption [11,12].

Traditional culture-based methods for isolating and enumerating *V. parahaemolyticus *from seafood samples involve the most probable number (MPN) technique [13]. Although widely used, such methods are labor-intensive and time-consuming (4-7 days). Molecular-based methods such as DNA probe hybridization and PCR assays have been developed for *V. parahaemolyticus *and yielded rapid and specific results [14-18]. However, the probe hybridization procedure and the gel electrophoresis technique used to analyze PCR amplicons are tedious and time-consuming. Recently, several real-time PCR assays have been developed for the detection of *V. parahaemolyticus *with increased speed and sensitivity [12,19-21]. Nonetheless, these assays require a dedicated real-time PCR machine, which is rather expensive and not yet widely available.

Loop-mediated isothermal amplification (LAMP), a novel DNA amplification technique invented in 2000 [22], has since been applied in detecting many bacterial and viral agents [23-26]. Because the LAMP assay was carried out under isothermal conditions, a simple heater that maintains a constant temperature (60-65°C) is sufficient. LAMP assays were reported to be highly specific, sensitive, rapid, and cost-effective [23-26]. Very recently, LAMP was adopted to detect *V. parahaemolyticus *and yielded promising results [11]. However, in this LAMP assay, primers were designed to target the *V. parahaemolyticus *thermolabile hemolysin gene (*tlh*), and an increasing number of hemolysin gene sequences with various levels of identity to this gene have been described in other vibrios such as *Vibrio harveyi *and *Vibrio campbellii *[27,28]. A search of the GenBank also revealed significant homologies among these hemolysin genes http://www.ncbi.nih.gov/BLAST. Additionally, Croci et al. [29] evaluated several PCR assays for the identification of *V. parahaemolyticus *by targeting different genes. Among 48 *V. parahaemolyticus *and 115 other *Vibrio *spp. strains examined, the two *tlh*-based PCR protocols [13,14] obtained 100% inclusivity but had 50% and 91% exclusivity, respectively. In contrast, a *toxR*-based PCR assay [18] simultaneously evaluated in the same study achieved 100% for both inclusivity and exclusivity [29].

The *toxR *gene was initially described in *V. cholerae *as the regulatory gene for the cholera toxin and other virulence determinants [30], and was subsequently found in *V. parahaemolyticus *[31]. Although present in many *Vibrio *spp., a membrane "tether" region within the coding sequence of *toxR *possesses significant heterogeneity and could be used to distinguish various *Vibrio *species [32]. The objective of this study was to develop a highly specific and sensitive *toxR*-based LAMP assay for the detection of *V. parahaemolyticus *in raw oyster samples.

## Results

### Specificity of the LAMP assay

The *V. parahaemolyticus toxR-*based LAMP assay run on two platforms by using either a real-time PCR machine or a real-time turbidimeter successfully detected 36 *V. parahaemolyticus *strains while showing negative results for 39 non- *V. parahaemolyticus *strains (Table 1), indicating that the *toxR-*based LAMP assay was highly specific. On the real-time PCR platform, mean cycle threshold (*Ct*; cycles when fluorescence signals reach 30 units) values for the 36 *V. parahaemolyticus *clinical and environmental strains ranged between 13.58 and 23.95 min, with an average of 17.54 ± 2.27 min. The melting temperatures (Mt) for these strains consistently fell between 81.25 and 82.55°C, with an average Mt of 81.97 ± 0.25°C. For the 39 non- *V. parahaemolyticus *strains, no *Ct *value was obtained, with melting curve analysis showing no peaks, suggesting no amplification occurred.

**Table 1:** Bacterial strains used in this study

Strain group	Strain ID and serotype ^*a*^	Source and reference
*V. parahaemolyticus*	ATCC 17802; O1:K1	Shirasu food poisoning, Japan
(*n *= 36)	ATCC 27969	Blue crab, Maryland
	ATCC 33847	Gastroenteritis, Maryland
	ATCC 49529; O4:K12	Feces, California
	CT-6636; O3:K6	Clinical, Connecticut
	M350A; O5	Oyster, Washington
	NY477; O4:K8	Oyster, New York
	TX-2103; O3:K6	Clinical, Texas
	8332924; O1:K56	Oyster, Gulf of Mexico
	83AO8757	Clinical, feces
	83AO9148	Clinical, feces
	83AO9756; O4:K12	Clinical, feces
	84AO1516; O4:K12	Clinical, feces
	84AO4226	Clinical, feces
	916i, 916e, 541-0-44c, V68, V69, V154, V155, V166	Oyster, Gulf, Louisiana [10]
	V5, V15, V16, V32, V38, V39, V50, V86, V150, V426, V427, V428, V429, V430	Oyster, Retail, Louisiana [10]
*V. vulnificus*	ATCC 27562	Blood, Florida
(*n *= 18)	ATCC 29306	Corneal ulcer, Virginia
	ATCC 33815	Leg ulcer, Wisconsin
	ATCC 33816	Blood, Alaska
	C7184	Thumb drainage, Texas [39]
	1003	Wound, Louisiana [40]
	1006	Blood, Louisiana [40]
	WR1	Sea water, Washington
	515-4C2	Oyster, California
	541-0-84c	Gulf oyster, Louisiana [10]
	V373, V416, V578, V598	Retail oyster, Louisiana [10]
	132A1, 132T5, 212B6, 342E3	Gulf oyster, Louisiana^*b*^
Other *Vibrio *spp. (*n *= 10)		
*Vibrio alginolyticus*	ATCC 17749	Spoiled horse mackerel, Japan
	ATCC 33787	Seawater, Hawaii
*Vibrio cholerae*	ATCC 14035; O:1	United Kingdom
*Vibrio cincinnatiensis*	ATCC 35912	Blood/cerebrospinal fluid, Ohio
*Vibrio fluvialis*	ATCC 33809	Human feces, Bangladesh
*Vibrio harveyi*	ATCC 14126	Dead amphipod, Massachusetts
	ATCC 35084	Brown shark, Maryland
*Vibrio mimicus*	ATCC 33653	Human ear, North Carolina
	ATCC 33655	Feces, Tennessee
*Vibrio natriegens*	ATCC 14048	Salt marsh mud, Georgia
Non-*Vibrio *spp. (*n *= 11)		
*Campylobacter jejuni*	81-176	Human
*Enterobacter aerogenes*	ATCC 13048	Sputum, South Carolina
*Enterococcus faecalis*	ATCC 29212	Urine
*Escherichia coli*	ATCC 25922	Human
*Listeria monocytogenes*	ATCC 13932; 4b	Spinal fluid, Germany
*Pseudomonas aeroginosa*	ATCC 27853	Human blood
*Salmonella enterica *	LT2; Typhimurium	Unknown
*Shigella flexneri*	ATCC 12022; 2b	Unknown
*Shigella sonnei*	ATCC 25931	Human feces, Panama
*Staphylococcus aureus*	ATCC 29213	Wound
*Streptococcus pneumoniae*	ATCC 49619; type 59	Sputum, Arizona

^*a *^ATCC, American Type Culture Collection, Manassas, VA.

^*b *^Isolated from three Louisiana coastal locations (designated as 132, 212, and 342) between 2006-2007.

On the real-time turbidimeter platform, time threshold (*Tt*; time when turbidity values reach 0.1) values for the 36 *V. parahaemolyticus *clinical and environmental strains ranged from 28.3 to 33.5 min with an average of 31.13 ± 1.67 min. For the 39 non- *V. parahaemolyticus *strains, no *Tt *value was obtained, indicating negative results for *V. parahaemolyticus toxR*-based LAMP assay.

Similarly, no false positive or false negative results for the 75 bacterial strains were observed by PCR using two primer sets, F3/B3 and *toxR*-PCR primers (Table 2), indicating good specificity.

**Table 2:** LAMP and PCR primers used in this study to detect *Vibrio parahaemolyticus*

Primer name	Sequence (5'-3')	Position^*a*^	Amplicon size (bp)	Reference
F3	TTGGATTCCACGCGTTAT	528-545	Ladder-like bands for LAMP; 183 bp for F3/B3 PCR	This study
B3	CGTTCAATGCACTGCTCA	693-710		
FIP	TGAGATTCCGCAGGGTTTGTAA TTATTTTTGGCACTATTACTACCG	587-608 (F1c) 547-570 (F2)		
BIP	GTTCCGTCAGATTGGTGAGTATC TAGAAGGCAACCAGTTGTT	609-631(B1c) 673-691(B2)		
Loop	AGAACGTACCAGTGATGACACC	632-653		
toxR-F	GTCTTCTGACGCAATCGTTG	453-472^*b*^	367^*b*^	[18]
toxR-R	ATACGAGTGGTTGCTGTCATG	799-819^*b*^		

^*a *^The positions are numbered based on the coding sequence of *V. parahaemolyticus *strain AQ3815 *toxR *gene [GenBank: L11929].

^*b *^Differences in primer positions and amplicon size were noted from those originally published after reanalysis of the sequences.

### Sensitivity of the LAMP assay

**Figure 1:**
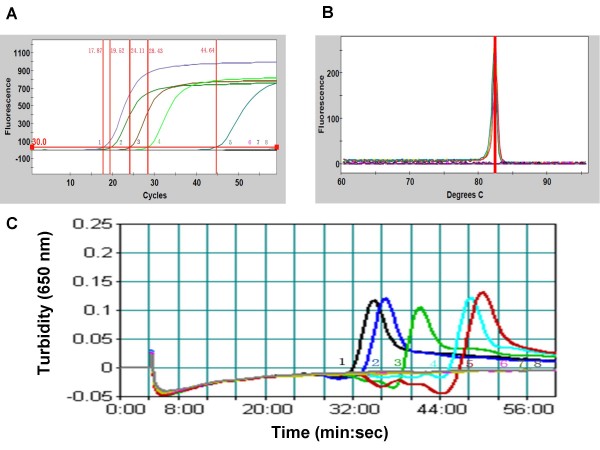
**Sensitivity of the LAMP assay when detecting *Vibrio parahaemolyticus *ATCC 27969 in pure culture**. (A) A representative optic graph generated using the real-time PCR machine; (B) Corresponding melting curve analysis for samples in (A); (C) A representative turbidity graph generated using the real-time turbidimeter. Samples 1-7 corerspond to serial 10-fold dilutions of *V. parahaemolyticus *ATCC 27969 cells ranging from 4.7 × 10^5 ^to 4.7 × 10^-1 ^CFU/reaction; sample 8 is water.

Figure 1 presents sensitivity of the *toxR*-based LAMP assay when testing 10-fold serial dilutions of *V. parahaemolyticus *ATCC 27969 DNA templates. A representative optic graph and corresponding melting curve analysis for the real-time PCR platform and a representative turbidity graph for the real-time turbidimeter platform are shown in Figure 1A-1C, respectively. On the real-time PCR platform, for templates ranging in concentration from 4.7 × 10^5 ^to 4.7 × 10^1 ^CFU per reaction tube, the average *Ct *values of six repeats ranged from 17.35 to 40.72 min, with melting temperatures consistently falling at around 83°C. No amplification was obtained for the 4.7 CFU and 4.7 × 10^-1 ^CFU templates. Therefore, the detection limit of the *toxR*-based LAMP assay run in a real-time PCR machine was approximately 47 CFU per reaction. In the real-time turbidimeter platform, the average *Tt *values fell between 34.43 and 49.07 min for templates ranging from 4.7 × 10^5 ^to 4.7 × 10^2 ^CFU per reaction tube. In two out of six repeats, amplification of the 4.7 × 10^1 ^CFU template occurred (Figure 1C). Therefore, the lower limit of detection for turbidity-based real-time LAMP assay was 47-470 CFU per reaction.

The two PCR assays used to test the same set of *V. parahaemolyticus *ATCC 27969 templates by using F3/B3 and *toxR*-PCR primers had the same level of sensitivity, approximately 4.7 × 10^3 ^CFU per reaction tube (data not shown), i.e., up to 100-fold less sensitive than the *toxR*-based LAMP assay.

### Quantitative capability of LAMP for detecting *V. parahaemolyticus *in pure culture

**Figure 2:**
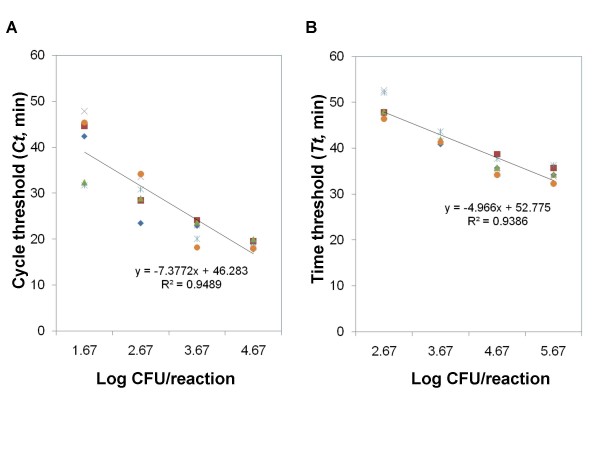
**Standard curves generated when detecting *Vibrio parahaemolyticus *ATCC 27969 in pure culture**. (A) Based on six independent repeats in a real-time PCR machine; (B) Based on six independent repeats in a real-time turbidimeter.

Figure 2 shows the standard curves generated when detecting *V. parahaemolyticus *ATCC 27969 in pure culture based on six independent repeats in both real-time PCR machine (Figure 2A) and a real-time turbidimeter (Figure 2B). On the real-time PCR platform, the correlation coefficient (*r*^2^) was calculated to be 0.95. When run in the real-time turbidimeter platform, the *toxR*-based LAMP assay had an *r*^2 ^value of 0.94.

### Detection of *V. parahaemolyticus *cells in spiked oysters

**Figure 3:**
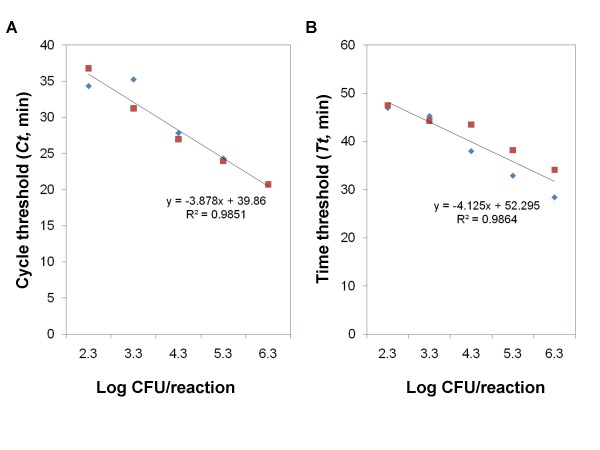
**Standard curves generated when testing *Vibrio parahaemolyticus *ATCC 27969 in spiked oysters**. Three sets of independent spiking experimetns were performed, and the LAMP reactions were repeated two times for each inoculation set. The data shown are for the inoculation set 3 ranging from 1.1 × 10^5 ^to 1.1 × 10^9 ^CFU/g. (A) The assay was run in a real-time PCR machine; (B) The assay was run in a real-time turbidimeter.

The sensitivity of detecting *V. parahaemolyticus *ATCC 27969 cells in spiked oyster samples is shown in Table 3. In three independent spiking experiments, the *toxR*-based LAMP assay using the two platforms consistently detected down to 200 *V. parahaemolyticus *cells (i.e., 1.1 × 10^5 ^CFU/g) in spiked oyster samples without enrichment. However, for the two PCR assays using F3/B3 and *toxR*-PCR primers, the lowest detection limit achieved was 1.1 × 10^6 ^CFU/g and 1.1 × 10^7 ^CFU/g, which were up to 100-fold less sensitive than that of the *toxR*-based LAMP assay. Standard curves (Figure 3) generated for the quantitative detection of *V. parahaemolyticus *cells in spiked oyster samples had an *r*^2 ^value of 0.99 for both real-time LAMP platforms.

**Table 3:** Comparison of quantitatively detecting *Vibrio parahaemolyticus *ATCC 27969 in spiked oysters by using the *toxR*-based LAMP assay in two platforms and PCR^a^

Rep.	Levels of spiking (CFU/g)	Amount of cells^*b *^(CFU/rxn)	LAMP			PCR	
			
			Fluorescence-based		Turbidity-based	F3/B3	*toxR*
					
			*Ct *(min)	Mt (°C)	*Tt *(min)		
1	5.6 × 10^8^	1.0 × 10^6^	20.61 ± 2.04	82.16 ± 0.05	31.2 ± 2.97	+	+
	5.6 × 10^7^	1.0 × 10^5^	22.02 ± 2.04	81.36 ± 1.20	35.3 ± 1.13	+	+
	5.6 × 10^6^	1.0 × 10^4^	25.26 ± 0.56	81.87 ± 0.10	42.55 ± 2.2	+	+
	5.6 × 10^5^	1.0 × 10^3^	**34.58 ± 2.25**	**82.45 ± 0.23**	**52.45 ± 2.75**	+	-
	5.6 × 10^4^	1.0 × 10^2^	-	-	-	-	-
	5.6 × 10^3^	10	-	-	-	-	-
2	1.7 × 10^8^	3.1 × 10^5^	21.78 ± 0.59	82.41 ± 0.11	29.4 ± 0.85	+	+
	1.7 × 10^7^	3.1 × 10^4^	23.68 ± 0.16	82.25 ± 0.10	33.25 ± 0.35	+	+
	1.7 × 10^6^	3.1 × 10^3^	29.08 ± 0.45	82.60 ± 0.34	40.4 ± 4.67	+	-
	1.7 × 10^5^	3.1 × 10^2^	**31.77 ± 2.23**	**82.50 ± 0.18**	**47.7 ± 1.27**	-	-
	1.7 × 10^4^	31	-	-	-	-	-
	1.7 × 10^3^	3.1	-	-	-	-	-
3	1.1 × 10^9^	2.0 × 10^6^	20.74 ± 0.03	82.48 ± 0.01	31.25 ± 4.02	+	+
	1.1 × 10^8^	2.0 × 10^5^	24.14 ± 0.24	82.37 ± 0.05	35.55 ± 3.73	+	+
	1.1 × 10^7^	2.0 × 10^4^	27.42 ± 0.60	82.48 ± 0.11	40.75 ± 3.88	+	+
	1.1 × 10^6^	2.0 × 10^3^	33.26 ± 2.84	82.50 ± 0.26	44.8 ± 0.7	+	-
	1.1 × 10^5^	2.0 × 10^2^	**35.57 ± 1.73**	**82.65 ± 0.09**	**47.25 ± 0.35**	-	-
	1.1 × 10^4^	20	-	-	-	-	-

Bolded are detection limits by each assay.

^*a *^For each independently prepared template, two times of LAMP reactions were performed and the data presented are means ± standard deviations for the 2 LAMP repeats.

^*b *^CFU/reaction was calculated by using CFU/g × 0.09 g/ml × 10 × 2 × 10^-3^, i.e., CFU/g × 1.8 × 10^-3^.

## Discussion

In this study, we designed a set of five LAMP primers to specifically target the *V. parahaemolyticus toxR *gene, a gene previously shown to possess better specificity for *V. parahaemolyticus *detection by PCR than other target genes, such as *tlh *and *gyrB *[29]. We also developed real-time LAMP assays using two platforms - a real-time PCR machine and a real-time turbidimeter to quantitatively detect *V. parahaemolyticus *in pure culture and spiked oyster samples. This is the first report demonstrating the efficacy of a *toxR*-based LAMP assay for detecting *V. parahaemolyticus *in oysters.

The LAMP primers were selected from regions of the *V. parahaemolyticus toxR *gene coding sequence that are highly specific to *V. parahaemolyticus *[18,32]. The five primers (F3, B3, FIP, BIP, and loop) targeted seven regions of *V. parahaemolyticus toxR *(Table 2), providing additional levels of specificity compared to PCR primers (targeting two regions). Among a total of 36 *V. parahaemolyticus *and 39 non-*V. parahaemolyticus *strains tested, the *toxR*-based LAMP assay run on both real-time platforms obtained 100% inclusivity and 100% exclusivity. This level of specificity was the same as that of two *toxR*-based PCR assays evaluated simultaneously in this study and that of a *tlh*-based LAMP assay developed by Yamazaki et al. [11]. Future pairwise comparison of the two LAMP assays (*toxR*-based and *tlh*-based) using an extensive collection of *Vibrio *strains as done previously for PCR [29] would be desired to further evaluate the performance of the two LAMP assays on both inclusivity and exclusivity.

When comparing the sensitivity of LAMP with PCR, the *toxR*-LAMP assays were able to detect 47-470 *V. parahaemolyticus *cells per reaction tube, in contrast to 4.7 × 10^3 ^cells for *toxR*-PCR. Similarly, the *tlh*-based LAMP assay for *V. parahaemolyticus *was reported to be 10-fold more sensitive than PCR, with a detection limit of 2 CFU per reaction for LAMP [11]. In a recent report on the detection of pathogenic *V. parahaemolyticus *by targeting the *tdh *gene, both LAMP and PCR were capable of detecting less than 1 CFU of TDH-producing *V. parahaemolyticus *in a reaction tube, although for different serotypes tested, slight difference in terms of sensitivity was observed [33]. Additionally, several studies on the detection of other *Vibrio *spp. also found LAMP to be 10-fold more sensitive than PCR [23,34,35].

Running the *toxR*-LAMP assay in a real-time PCR machine consistently achieved a lower limit of detection of 47 cells per reaction, whereas in a real-time turbidimeter, a detection limit of 47 cells was only occasionally achieved (2 out of 6 attempts). In addition, the average time to positive results as indicated by *Ct *(17.54 min) for the real-time PCR platform was markedly shorter than that of the real-time turbidimeter platform as indicated by *Tt *(31.13 min), suggesting that the real-time LAMP assay based on fluorescence was faster and slightly more sensitive than that based on turbidity. This finding agrees with a previous study which reported that a fluorescent intercalation dye (YO-PRO-1)-based real-time LAMP was 10-fold more sensitive and faster than a turbidimetry real-time LAMP [36]. However, in that report, the fluorescence-based LAMP assay was found to generate anomalous and irreproducible results in low-concentration templates (less than 1 × 10^3 ^copies), which could be due to the effect of the intercalating dye on DNA amplification efficiency [36].

In this study, we chose SYTO-9 as the intercalating dye for the real-time PCR platform instead of the commonly used real-time PCR dye SYBR Green I. Based on a previous study [37] comparing the use of these two dyes in real-time PCR, SYTO-9 was found to generate highly reproducible DNA melting curves over a broader range of dye concentrations than SYBR Green I and was far less inhibitory. We also evaluated the use of EvaGreen (Biotium, Hayward, CA) as the intercalating dye on the real-time PCR platform for LAMP, but found it to be inhibitory for LAMP amplifications (data not shown).

The strong linear correlation (*r*^2 ^= 0.94-0.99) between the number of *V. parahaemolyticus *cells in the LAMP reaction and the associated *Ct *or *Tt *values over a dynamic range of template concentrations (10^1 ^to 10^6 ^cells) illustrates the quantitative capability of the *toxR*-based real-time LAMP assays when detecting this organism in both pure culture and spiked oysters. Very few reports have examined the quantitative ability of LAMP. One study monitoring ammonia-oxidizing bacteria using LAMP also reported it to possess good quantitative capability between 1 × 10^4 ^and 1 × 10^10 ^DNA copies [36].

In spiked oyster samples, we found the detection limit of the *toxR*-based LAMP assay to be 200 *V. parahaemolyticus *cells per reaction, which translates to 1.1 × 10^5 ^cells per gram of oyster sample. In contrast, the detection limit of the *tlh*-based LAMP in spike shrimp samples was reported to be 5.3 × 10^2 ^CFU/g (2 CFU/reaction) [11]. The U.S. Food and Drug Administration requires that all postharvest-processed oysters have lower than 30 MPN/g of either *V. vulnificus *or *V. parahaemolyticus *[38]. This indicates that without enrichment, DNA amplification assays such as LAMP, although potentially quantitative, lack the needed sensitivity when applied to food samples [23]. Therefore, combining MPN overnight enrichment [19] or pre-enrichment for 6 h [33] with LAMP or other DNA amplification assays is a desirable approach to achieve the needed sensitivity.

When testing spiked oyster samples, we observed the time to positive samples (*Ct *for the real-time PCR platform and *Tt *for the real-time turbidimeter) was delayed several minutes compared to pure culture samples and the detection limit was higher (200 *V. parahaemolyticus *cells in oyster samples vs. 47 cells in pure culture). Nonetheless, no extensive sample preparation other than homogenization and two simple centrifugation steps was required. This significantly reduced the total assay time. Combined with less than 1 h for the real-time LAMP assay, the complete LAMP detection system was markedly faster than either PCR or conventional methods.

## Conclusions

The *toxR*-based real-time LAMP assay developed in this study was a highly specific, sensitive, and rapid method for the detection of *V. parahaemolyticus *in oysters. Future testing with natural or commercial oyster samples is desired to further evaluate the efficacy of the assay in detecting *V. parahaemolyticus *in oysters in a field setting.

## Methods

### Bacterial strains and DNA templates preparation

Strains used in this study (Table 1) were maintained in Luria-Bertani broth (BD Diagnostic Systems, Sparks, MD) containing 30% glycerol at -80°C. *V. parahaemolyticus *ATCC 27969, originally isolated from blue crab hemolymph was used for sensitivity testing. Additional 35 *V. parahaemolyticus *clinical and environmental strains and 39 non- *V. parahaemolyticus *strains were used to evaluate assay specificity. All *Vibrio *strains were routinely cultured using trypticase soy agar or broth (TSA or TSB; BD Diagnostic Systems) supplemented with 2% NaCl at 35°C overnight. Non-*Vibrio *strains were grown on Luria-Bertani agar or blood agar (BD Diagnostic Systems).

To prepare DNA template, a single bacterial colony grown on appropriate agar plates was suspended in 500 μl of TE buffer (10 mM Tris, pH 8.0; 1 mM EDTA; Sigma-Aldrich, St. Louis, MO) and heated at 95°C for 10 min in a dry heating block. The crude cell lysate was centrifuged at 12,000 *g *for 2 min and the supernatant was stored at -20°C until use.

### LAMP primers and reaction conditions

The *V. parahaemolyticus toxR *gene [GenBank: L11929] was used as the target for LAMP primer design. Five primers, two outer (F3 and B3), two inner (FIP and BIP), and one loop (Loop) which recognized seven distinct regions of the target sequence were designed using the PrimerExplorer software version 4 (Fujitsu Limited, Japan; http://primerexplorer.jp/e. Oligonucleotide sequences and locations of the primers are shown in Table 2. The primers were synthesized by Invitrogen (Carlsbad, CA).

The LAMP reaction mix in a 25 μl total volume consisted of the following: 1 × Thermo buffer, 6 mM of MgSO_4_, 0.8 M of betaine (Sigma-Aldrich), 1.4 mM of deoxynucleotide triphosphate (dNTP), 0.2 μM of each outer primer (F3 and B3), 1.6 μM of each inner primer (FIP and BIP), 0.8 μM of the loop primer, 8 U of *Bst *DNA polymerase (New England Biolabs, Ipswich, MA), and 2 μl of DNA template. Additionally, 0.4 μM of SYTO-9 green fluorescent dye (Invitrogen) was added when the LAMP reaction was carried out in a real-time PCR machine as described below.

Two platforms were used to run the LAMP reactions. On the first platform, a real-time PCR machine (SmartCycler II System; Cepheid, Sunnyvale, CA) was used and the SYTO-9 green fluorescent dye was added. The assay was conducted at 63°C for 1 h. Fluorescence readings were acquired every 60 s using the FAM channel (excitation at 450-495 nm and detection at 510-527 nm), followed by melting curve analysis from 63°C to 96°C with 0.2°C increment per second. The fluorescence threshold unit was set to be 30. On the second platform, the LAMP reaction was carried out in a Loopamp real-time turbidimeter (LA-320C; Teramecs, Kyoto, Japan) at 63°C for 1 h and terminated at 80°C for 5 min. Turbidity readings at 650 nm were obtained real-time and a turbidity threshold value of 0.1 was used. A negative control was included for each LAMP run.

### PCR

As a comparison, two sets of PCR reactions were performed, one using LAMP outer primers (F3 and B3) and the other one using the *toxR*-PCR primers (Table 2) published previously [18]. Each PCR mix in a 25 μl total volume contained 1 × PCR buffer, 0.2 mM of each dNTP, 1.5 mM of MgCl_2_, 0.5 μM of each forward and reverse primer, 0.625 U of GoTaq Hot Start Polymerase (Promega, Madison, WI), and 2 μl of DNA template. The PCR reactions were conducted using initial denaturation at 95°C for 5 min followed by 30 cycles of denaturation at 94°C for 1 min, primer annealing at 60°C (50°C for F3/B3 primers) for 1 min, extension at 72°C for 1 min, and a final extension at 72°C for 7 min in a Bio-Rad C1000 Thermal Cycler (Hercules, CA). Aliquots (10 μl) of PCR products were analyzed by electrophoresis on 1.5% agarose gel containing ethidium bromide, and visualized under UV light. Gel images were documented by a Gel Doc XR system (Bio-Rad).

### LAMP specificity and sensitivity

Seventy-five bacterial strains (Table 1) were used to determine the LAMP specificity. DNA templates were made from fresh overnight bacterial cultures and aliquots (2 μl) were subjected to both LAMP and PCR amplifications. Specificity tests were repeated twice.

To determine LAMP sensitivity, serial 10-fold dilutions (*ca. *10^8 ^CFU/ml to extinction) of a mid-log phase *V. parahaemolyticus *ATCC 27969 culture grown in TSB were prepared in phosphate buffered saline (PBS; BD Diagnostic Systems) and quantified using the standard plating method. DNA templates were prepared from each dilution by the boiling method described above and aliquots (2 μl) were subjected to both LAMP and PCR amplifications. Sensitivity tests were repeated six times and the lower limits of detection (CFU/reaction) were reported. Standard curves were generated by plotting *Ct *(cycle threshold; for the real-time PCR platform) or *Tt *(time threshold; for the real-time turbidimeter platform) values against log CFU/reaction and the linear regression was calculated using the Microsoft Excel Software (Seattle, WA).

### LAMP testing in experimentally inoculated oyster samples

Oyster samples were obtained from local seafood restaurants and determined to be *V. parahaemolyticus*-negative as described previously [10]. Oyster samples were processed following a previous study with slight modifications [11]. Briefly, 25 g of oyster sample was mixed with 225 ml of alkaline peptone water (APW; BD Diagnostic Systems) and homogenized in a food stomacher (Model 400; Tekmar Company, Cincinnati, OH) for 90 s to generate 1:10 oyster in APW homogenate. Serial 10-fold dilutions of a mid-log phase *V. parahaemolyticus *ATCC 27969 culture were prepared in PBS as described above. Aliquots (100 μl) of each dilution were inoculated into 900 μl of the 1:10 oyster in APW homogenate. The spiked oyster samples were mixed well and centrifuged at 900 *g *for 1 min to remove oyster tissues. The supernatants were transferred to a fresh tube and centrifuged at 10,000 *g *for 5 min to pellet bacterial cells. After removing the supernatants, pellets were resuspended in 100 μl of TE and boiled for templates as described above. Aliquots (2 μl) of the supernatant were used for both LAMP and PCR amplifications. The spiked oyster sensitivity tests were repeated three times and the lower limits of detection (CFU/g) were reported. Standard curves were generated similarly as in pure culture sensitivity testing.

## Authors' contributions

SC carried out the LAMP and PCR assays, conducted data analysis, and drafted the manuscript; SC and BG conceived of the study and participated in its design. BG coordinated the study and helped to finalize the manuscript. Both authors read and approved the final manuscript.
